# Deep learning approaches for conformational flexibility and switching properties in protein design

**DOI:** 10.3389/fmolb.2022.928534

**Published:** 2022-08-10

**Authors:** Lucas S. P. Rudden, Mahdi Hijazi, Patrick Barth

**Affiliations:** Institute of Bioengineering, Swiss Federal Institute of Technology (EPFL), Lausanne, Switzerland

**Keywords:** deep learning, protein design, generative models, protein flexibility, protein switches

## Abstract

Following the hugely successful application of deep learning methods to protein structure prediction, an increasing number of design methods seek to leverage generative models to design proteins with improved functionality over native proteins or novel structure and function. The inherent flexibility of proteins, from side-chain motion to larger conformational reshuffling, poses a challenge to design methods, where the ideal approach must consider both the spatial and temporal evolution of proteins in the context of their functional capacity. In this review, we highlight existing methods for protein design before discussing how methods at the forefront of deep learning-based design accommodate flexibility and where the field could evolve in the future.

## Introduction

By interacting with substrates, performing precise chemical reactions, and transducing signals, proteins directly govern a wide range of regulatory functions in living cells. Consequently, *in silico* protein design, where a protein is either re-engineered from a native template or *de novo* designed, offers a direct route to addressing a wide range of complex bioengineering issues (Mahendran et al., 2020; Sterner and Sterner, 2021) without expensive and time-consuming experimental screening. *De novo* design can, in principle, facilitate the programming of any desired function, making it highly versatile over re-engineering, which is more restricted by the native protein fold. However, pure *de novo* protein design is often more challenging than re-engineering. It requires careful consideration of the optimal binding site that confers the desired function, and the active fold state of the designed protein must be both thermodynamically stable and kinetically accessible along a folding pathway. The inherent flexibility of proteins further exacerbates this complexity from a local side-chain to global scale, where multiple conformational states can be crucial for function. Switching between states can be triggered by external stimuli such as ligand binding. Thus, the design of any function that requires some internal motion such as molecular transport, allosteric regulation, and mechanotransduction, must carefully consider the coupling between the stimuli and switching of a protein’s occupied fold state and subsequent functional capacity.

Over the last 3 years, there has been a shift in the paradigm in the biophysical study of proteins, with the application of deep learning (DL) methods for structure prediction far outperforming traditional physics-based methods (Pakhrin et al., 2021). Broadly, DL is used to process unstructured data to learn underlying descriptors of that data (features) that can then be exploited for either generative or classification purposes. Some data, such as discrete variables, can be projected into a higher dimension (embedding) such that features that are more alike are closer in the embedding space, enabling more meaningful learning of relationships. By leveraging the many layers of a neural network, DL can learn complex and non-linear relationships to map the raw input into some low dimensional latent space that describes the data. The power of DL, and machine learning in general, is in backpropagation, where the error between the output of a network, such as in a classification task, is connected directly to the input of the network in an end-to-end fashion—with the weights connecting nodes between layers adjusted based on the overall gradient.

Protein structure prediction methods such as AlphaFold2 (Jumper et al., 2021) and RoseTTAfold (Baek et al., 2021), rely on a multiple sequence alignment (MSA) to learn an evolution-based history of residue contacts, working in harmony with a pairwise feature map that encodes information about residue relationships. Predicted structures represent the most likely state occupied by a protein given the distribution of structural states present in the PDB training data and input MSA. Therefore, while local flexibility is inherently accounted for within these networks, conformational switching is (usually, see later) not. This represents a significant limitation in current structure prediction methods. Thus most conformational state-based design studies continue to rely on re-engineering existing proteins known to occupy multiple states (Alberstein et al., 2022).

**FIGURE 1:**
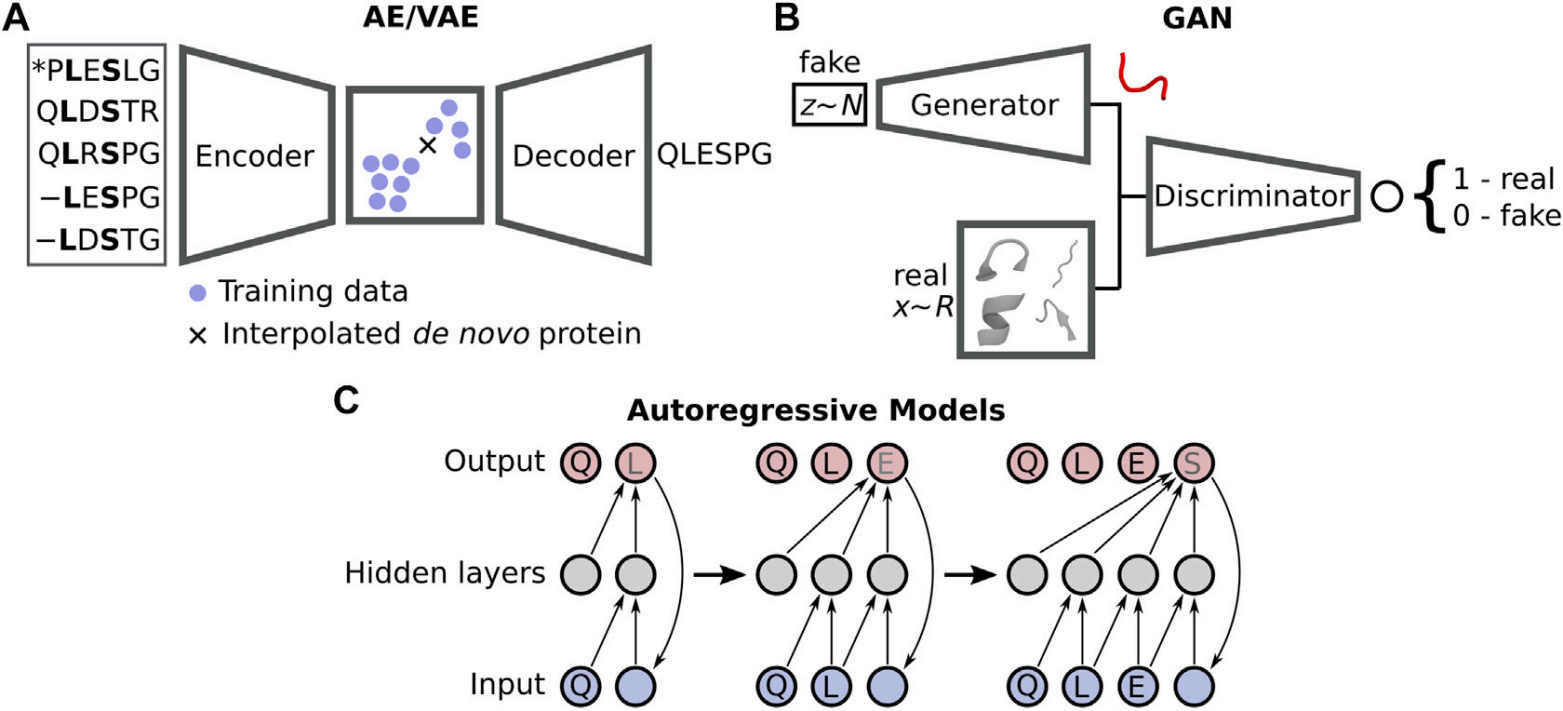
Three types of generative models are generally applied in DL-based protein design: **(A)** autoencoders/variational autoencoders (AE/VAEs), **(B)** generative adversarial networks (GANs), and **(C)** autoregressive models.

Despite the current limitations, the improvements gained by moving to DL-based prediction has motivated a similar change within the protein design community, with novel methods distancing themselves from the traditional design approaches such as Rosetta (Huang et al., 2011; Ollikainen et al., 2015; Bonet et al., 2018) and others (Röder and Wales, 2018) that rely on scoring functions describing physical energies. Numerous DL design strategies have recently emerged, broadly falling into two categories: sequence-(Wu et al., 2021) and structure-based design (Ovchinnikov and Huang, 2021). These employ what are known as generative neural networks, which create an underlying model that represents the distribution of the example training data. Interrogation of this model *via* interpolation in a constructed latent space yields plausible samples, i.e., non-native proteins. DL protein design chiefly uses one of three types of generative networks (Figure 1): autoencoders (AE) and closely related variational autoencoders (VAE) (Kingma and Welling, 2014), generative adversarial networks (GAN) (Goodfellow et al., 2014), and autoregressive likelihood models (Bengio et al., 2003). There are other generative networks yet to be directly applied to protein design (Bond-Taylor et al., 2021), but they have seen use in adjacent problems such as protein-protein interaction prediction (Gainza et al., 2019) and the modelling of protein dynamics (Noé et al., 2019). Both AEs and VAEs utilise an encoder to convert real features, e.g., coordinates, into a latent space representing either a transformation of the original data (AE) or a Gaussian distribution of the original data (VAE). A decoder is then used to sample this latent space, where interpolation between training samples yields plausible solutions, although this is more challenging for AEs as the latent space is non-regularised. GANs pit a generator network producing fake but realistic data against a discriminator, which attempts to decide if an input sample is real or not. The two compete, resulting in an iterative improvement of both the generator and discriminator. Autoregressive models, often used for Natural Language Processing, forecast future data samples based on historical context—such as the next amino acid in a sequence. We refer the reader to the recent review by Strokach and Kim (2022) where they discuss these models in extensive detail within the context of protein design.

**FIGURE 2:**
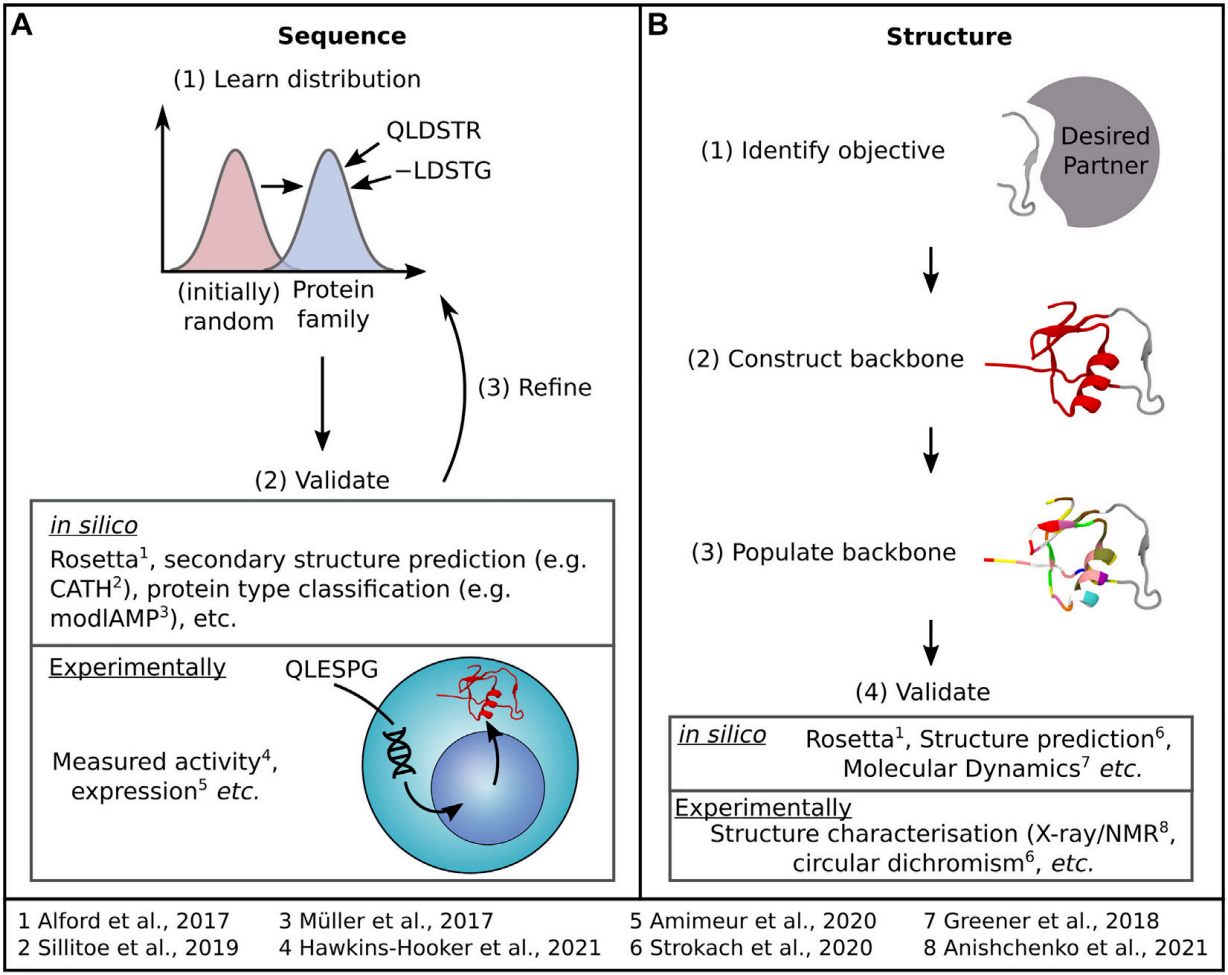
Most DL protein design methods tackle design as either a **(A)** sequence generation or **(B)** structure generation problem, each accompanied by the general process outlined here. Examples of both methods used to assess the quality of generated samples and specific DL protein design examples are also indicated.

Sequence design (Figure 2A) relies on learning a distribution of protein family sequences to sample new sequences that offer similar or improved functionality. Structure design (Figure 2B) begins with a design objective—such as a binding site fold and aims to generate a structure that supports that objective before populating the structure with a sequence. Much like in structure prediction, dealing with flexibility in these networks remains a challenge. Herein, we will briefly overview these current methods, summarised briefly in Table 1, before discussing how innovative approaches are considering the question of protein flexibility in the design of proteins and how we could better harness MSA data in protein design. For a more detailed insight into the latest advances in sequence and structure design DL methods, we refer the reader to recent reviews by Wu et al. (2021) and Ovchinnikov and Huang (2021), respectively.

**TABLE 1:** Summary of the key deep learning protein design methods discussed in this review, with their generation type and generative model type indicated by a *. ∼ in the structure design field suggests that some minor design coincides with sequence design. The design target of each method is also provided.

Method	Generation type		Generative model			Design target
	Sequence	Structure	VAE	GAN	Autoregressive	
Grisoni et al. (2018)	*				*	Antimicrobial peptides
Müller et al. (2018)	*				*	Membranolytic anticancer peptides
PepCVAE	*		*			Antimicrobial peptides
Hawkins-Hooker et al. (2021)	*		*			Luciferase enzymes
ProteinGAN	*			*		MDH-like enzymes
Greener et al. (2018)	*		*			Metalloproteins
Gupta and Zou (2019)	*			*		Antimicrobial peptides
Amimeur et al. (2020)	*			*		Human antibodies
Ingraham et al. (2019)	*				*	Non-specific
ProteoGAN	*			*		Non-specific
ProteinSolver	*				*	Non-specific
Anand et al. (2022)	*				*	Non-specific
Ig-VAE		*	*			Immunoglobulins
Anand et al. (2019)		*		*		Non-specific
Tischer et al. (2020)	*	∼	Inverted structure prediction model			Non-specific
Anishchenko et al. (2021)	*	*	Inverted structure prediction model			Non-specific
Norn et al. (2021)	*	∼	Inverted structure prediction model			Non-specific

## Sequence versus structure in deep learning protein design

### Sequence generation

Sequence design leverages available sequence data (Bateman et al., 2021) to learn statistical patterns that indicate function or folding stability (Wu et al., 2021). Networks are typically trained to learn the distribution of sequences in a desired protein family, from which new protein sequences can be extracted. Recurrent neural networks, a subclass architecture of autoregressive models, have been used to design antimicrobial and membranolytic anticancer peptides (Grisoni et al., 2018; Müller et al., 2018). PepCVAE constructs a latent space representing the distribution of known sequences for antimicrobial peptides, where interpolation within the space yields novel sequences (Das et al., 2018), and Hawkins-Hooker et al. (2021) recently included MSA data within VAE training to produce active luciferase enzymes. ProteinGAN has been similarly designed to produce active enzyme sequences (Repecka et al., 2021). While sequences generated through these methods have been identified as functional *in silico* (Alford et al., 2017; Müller et al., 2017; Sillitoe et al., 2019), they are not necessarily improvements on native proteins, and owing to the training method, any novel functionality is generally serendipitous. Attempts have been made to optimise sequences to improve functionality *via* biased training data in GANs (Gupta and Zou, 2019; Amimeur et al., 2020), and reinforcement learning (Angermüller et al., 2020), though these serve more as examples of functional optimisation than programming. Conditional learning, where data in pre-defined categories is used to train the network such that new samples can be generated based on those categories, is necessary to deliver fine-tuned programming. However, while sequence generative models can harness divergent sequences from the proteome to offer protein variants with novel functionality, conditional learning to control this functionality remains in its infancy. Greener et al. (2018)’s VAE was trained to produce sequences containing metal-binding sites based on the labelling of bound metal cofactors. Current efforts with a biased network training approach (Gupta and Zou, 2019; Amimeur et al., 2020) to introduce some programmability need to ensure a delicate balance between sequence diversity and the desired functional result (Linder et al., 2020). Ingraham et al. (2019) were able to design sequences using an autoregressive model conditioned on graphs of 3D structures, designing plausible sequences for protein folds outside the training data, providing an example of more targeted sequence functional design given the relationship between structure and function. Kucera et al. (2022)’s GAN offers one of the first examples of a function-specific conditional general sequence generation method. Trained on labels of the hierarchical Gene Ontology, their network was able to produce a wide variety of proteins with distinct functional properties based on the input label or labels, including mixed labels absent in the training data. Nevertheless, improving the functional specificity, e.g., activation from a specific ligand, is a considerably more difficult task given the niche training set size. All sequence-based methods require significant validation, most relying on *in silico* methods such as peptide classifiers (Müller et al., 2017) outside the gold standard of experimental testing. Kucera et al. introduced a novel *in silico* validation metric based on ensuring sequence diversity, conditional consistency with the labels, and distributional similarity to try and address the absence of reliable evaluation metrics. Arguably the most effective *in silico* validation method of structure prediction may prove challenging. Generated *de novo* sequences featuring high conformational entropy versus any native sequence may not be structurally verifiable with conventional or DL-based protein folding methods such as AlphaFold2, although new orphan-protein structure prediction DL methods are emerging that could address this (Chowdhury et al., 2021; Wang et al., 2022).

### Structure generation

The workflow of structure generation typically follows four stages: 1) formulation of a design objective (e.g., a fold that confers desired binding), 2) the generation of coordinates that support the fold, 3) sequence design to stabilise any generated structure, and 4) evaluation of generated designs, typically *via* Molecular Dynamics or Rosetta energy checks (Ovchinnikov and Huang, 2021). By considering the design objective from the first stage, structure generation already addresses one of the key limitations of sequence generation in that the specific functional outcome is used as a constraint in design. Stage 2 can be achieved with 1-3D data. 1D data typically describes local bond lengths, angles etc., and non-local features such as interaction energies between residues (O’Connell et al., 2018; Wang et al., 2018); recurrent networks have already been applied in protein forcefield development (Greener and Jones, 2021) and could be extended to design. 2D pairwise matrices can leverage popular image classifiers or “deepfake” methods (Eguchi and Huang, 2020). Finally, the most challenging is 3D coordinate data, which is always unique to the input protein, unlike 1D or 2D basic descriptors such as contact maps, which share many common attributes (e.g., bond lengths) across the proteome, although there are examples of 3D DL structure generation (Eguchi et al., 2020). Exacerbating the complexity, unlike 1D and 2D data, 3D data is not rotationally invariant, necessitating careful treatment in design (Renaud et al., 2021). However, direct 3D design is end-to-end, i.e., the conditions and objectives are fully connected to the direct 3D output, meaning backpropagation occurs directly from a proposed structural solution to the input. In contrast, 1D and 2D data must be converted to 3D coordinate data in a separate stage outside the network. This is analogous to sequence design, where further validation is often required *in silico* through structure prediction. Therefore, while more challenging, direct 3D structure generation must approximately learn protein physics to produce reasonable structures, a highly generalisable property. Numerous approaches exist to tackle converting 1-2D maps to 3D (Anand and Huang, 2018), such as a decoder network featuring two discriminators able to handle GAN generated output without a ground truth and produce coordinates with the correct chirality (Anand et al., 2019). Stage 3 of structure generation commonly wield pre-existing sequence design methods to stabilise the backbone. For example, structural designs generated by the aforementioned GAN (Anand et al., 2019) and VAE have produced immunoglobulin specific backbones and SARS-CoV-2 binders (Eguchi et al., 2020) using standard Rosetta FastDesign (Bhardwaj et al., 2016) to fill the backbone. Thus, a limitation of current structure generation lies in its inability to include sequence and by extension side-chain interactions that stabilise protein structures during design. While not explicitly considering side-chain interactions, sequence generation, particularly those that leverage powerful transformer-based language models (Ferruz and Höcker, 2022), can identify potential relationships between individual amino acids that confer stability. ProteinSolver (Strokach et al., 2020) is a DL example of a backbone sequence populator, leveraging a graphical neural network that splits individual amino acids into nodes connected by edges that represent distance constraints to predict masked residue positions. Aside from an expanded training dataset, it improves on Ingraem et al. (2019)’s approach by considering both the successive and preceding residue identities during design. Trained on 72 million sequences corresponding to 80,000 unique structures, the network learnt the relationship between common structural and sequence motifs, ultimately providing *de novo* sequences for four stable protein folds absent in the training set. However, side-chain reconstruction was neglected within the network, which is crucial for determining thermodynamic stability. The authors instead relied on homology modelling of large, generated datasets for validation. In contrast, Anand et al. (2022) aimed to explicitly build side-chain conformers given a structural template and evaluate a full atomistic model using a conditional convolutional autoregressive neural network. Their approach iteratively samples amino acid types and rotamers at specific residue positions conditioned on the local chemical environment, producing sequences that satisfied the fold of a *de novo* TIM-barrel backbone (Huang et al., 2016b), indicating that their network had learnt something of the underlying physics that guides folding.

Directly comparing the two general strategies for protein design purposes, structure generation appears more versatile than sequence generation as the inclusion of functional objectives such as binding site folds enhances functional programmability (Gao et al., 2020). Indeed, the increased variety of features allows one to profit from more advanced techniques in Machine Learning. Furthermore, structure design is more generalisable, as demonstrated by Table 1, with most sequence generation methods requiring some specific protein family design target. However, structure generation methods must still undergo subsequent sequence design. This disconnect between structure and sequence is inherently problematic from a switchable state perspective, as to perform multi-state design on an ensemble of generated backbones is computationally expensive. The same disconnect is true in reverse of course, with the lack of explicit modelling in sequence generation hindering our ability to design conformational flexibility.

## Accounting for flexibility in deep learning protein design

**FIGURE 3:**
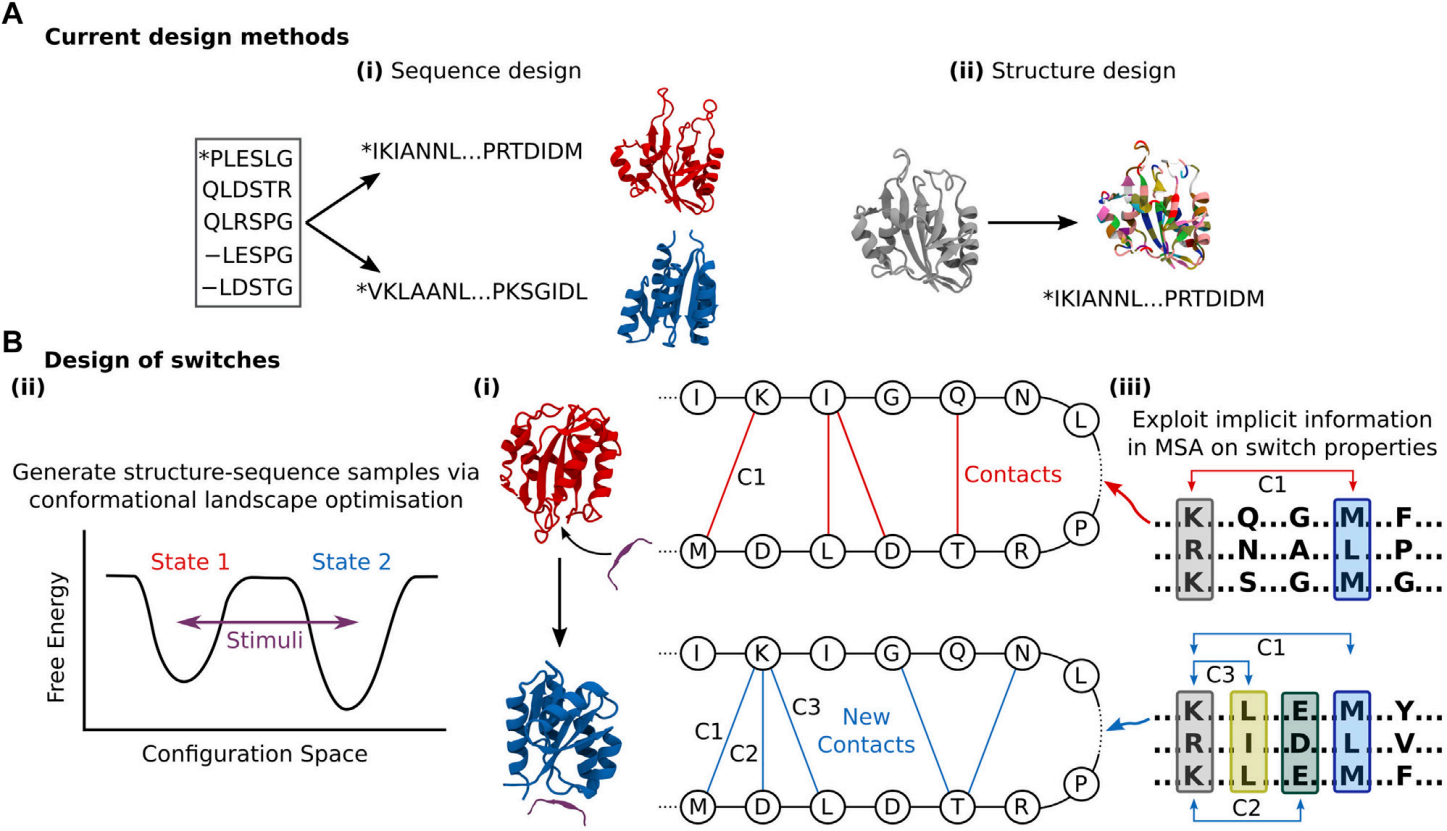
**(A)** Current design methods either: (i) Produce new sequences corresponding to some structure with limited design objective conditioning that could be leveraged for conformational flexibility design. (ii) Produce novel folds that confer some function that must be stabilised through sequence design. Both these approaches are inherently negligent of conformational flexibility. **(B)** (i) The general goal of DL-based protein switch design is to connect multiple structures to one sequence, with conformational perturbation triggered by some controlled signal. I.e., Given some stimuli (e.g., palatinate peptide), the contacts of a designed sequence in one state (red) shift given some new fold (blue), providing novel functional capacity. This could be achieved through (ii) Conformational landscape optimisation of multiple states given some design objective, similar to Norn et al., (iii) Harnessing of implicit relationships between sequence and multiple structures contained within MSA data, as demonstrated by del Alamo et al. (2022). Here, co-evolving residues (denoted in the coloured blocks) in two different low-depth MSAs make distinct contacts (shown as C1, C2, etc.,) that change the overall fold state.

Sequence generation effectively avoids the question of flexibility entirely by relying on pure bioinformatics, and thus cannot explicitly consider the flexibility problem. Yet, sequence generation methods can yield novel structural folds representative of hybridisation between homologous sequences that represent a spectrum of structural states. For instance, ProteinGAN produced distinct structural sequence motifs, validated by CATH (Sillitoe et al., 2019), suggesting that the network can learn generalised relationships between residues and produce sequences with increased structural diversity (Repecka et al., 2021). However, these sequences still only represent a single structural fold. Ultimately, even if proteins with purpose-built conformational flexibility or switchable state can be constructed, any functionality must be verified through further *in silico* validation (Nivedha et al., 2018; Chen et al., 2020), meaning programming function from sequence generation is not end-to-end differentiable. Analogous to structure prediction, structure generation methods inherently account for local side-chain flexibility owing to the ensemble of rotamer positions examined per residue during construction (Defresne et al., 2021). Yet there remains a conceptual gap to more extensive conformational flexibility. The sequence-structure design problem is still treated as a one-to-one mapping (Figure 3A), when in fact conformational selection requires concurrent exploration of sequence and structure design space given that a sequence can be connected to multiple fold states. Instead, during the sequence population stage of structure generation, the goal is primarily to stabilise the identified fold, and not offer perturbed structures which deviate from those produced by the network. Some exceptions exist; Tischer et al. (2020) created binding motifs within a discontinuous scaffold through the trRosetta structure prediction network (Yang et al., 2020) to tolerate larger backbone flexibility. They applied a loss function that rewarded both recapitulation of the input motif template and global structure stability, the latter facilitating some deviation from the inserted motif to ensure fold stability, thereby providing sequence and minor structure design. Norn et al. (2021) demonstrated that they could backpropagate gradients through trRosetta to generate sequences, exploring the sequence and structure space *via* optimisation of the conformational energy landscape towards one smooth funnelled state, thereby considering the global fold state and ensuring thermodynamic stability. Anishchenko et al. (2021) were able to generate completely *de novo* structure-sequence pairs by feeding random sequences into the trRosetta network, and performing an iterative Monte Carlo simulated annealing process to substitute individual amino acids randomly. By then re-predicting the distance and orientation maps from the network and accepting the substitution based on an increased Kullback–Leibler divergence, they transformed initially homogenous residue contact maps to ones with distinct structural features. Intriguingly, the similarity of the produced “hallucinated” sequences with native ones was very low, indicating the design of true *de novo* proteins. However, this process tended to neglect non-idealised structures, producing well-defined *α*-helices and *ß*-sheets connected by short loops. Long loops can be critical to function, from substrate binding, catalysis, and allosteric regulation. While it is noted that the loss function could be modified to retain specific sites such as binding interfaces (Tischer et al., 2020) or catalytic sites (Wang et al., 2021), whether this can be used to stabilise motifs such as binding loops remains to be seen. All three of these approaches that facilitate some structure design leverage inverted structure prediction models, as opposed to the direct generative models discussed above. While this makes intuitive sense given the inverted relationship between protein design and structure prediction, the consequence of this is that there is less control over the designed outputs, with the network acting as a black box. In addition to function conditional inputs, applying purpose-built generative models would allow for the specific application of powerful methods from the DL community.

While the networks by Norn et al. and Anishchenko et al. were able to learn something of the intimate relationship between structure and sequence, the adaptability of these methods towards purposeful conformational flexibility or switch design, where dual or even multiple conformational states are accessible by the same sequence is more challenging. Norn et al. optimised towards one clear funnelled state in the conformational landscape, while Anishchenko et al.’s network favoured selecting secondary structure elements that delivered global stability of a singular ground state, where any reshuffling into a second state would be energetically challenging. Yet, one of Anishchenko et al.’s designs did appear to adopt multiple monomeric conformations when tested experimentally, the authors attributing this switching behaviour to the lack of explicit side-chain representations in the modelling. While this is non-ideal for a monomeric protein without stimulus, the network has returned structure-sequence topologies able to adopt multiple conformations, albeit unintentionally. Adapting this approach to deliberately consider multiple states connected by a coherent path in the conformational landscape, while ambitious, could provide the means for switch design. Another potential solution lies in the greater exploitation of the known numerous states native proteins can occupy. In principle, multiple conformational landscapes should be connected to identical or evolutionary related sequences, the difference between them being perturbation by external stimuli (Figure 3B). AlphaFold2 recently demonstrated this *via* quaternary structure prediction (Ghani et al., 2021; Tsaban et al., 2022), where MSAs that included bound substrates returned different and accurate structural predictions versus the unbound MSA. In a peptide docking case, no MSA was necessary for the peptide, and the network could still predict conformational changes depending on the bound peptide (Tsaban et al., 2022). del Alamo et al. (2022) recently demonstrated they were able to predict multiple conformational states of transporters and GPCRs not present in the AlphaFold2 training data by reducing the depth of input MSA to AlphaFold2, indicating that while deep MSAs tend to relate to one fixed structure, stochastically sampled shallower MSAs are associated with a diversity of structural states. These works reveal that MSAs contain crucial underlying relationships that couple sequences with numerous plausible conformations (Wang and Barth, 2015), which could be leveraged for expanded functional capacity design. A sequence generation approach that also harnesses MSA data could recognise the divergence of structural states from phylogenetic trees of extensive protein families constructed using existing modern methods (Azouri et al., 2021). Here, the goal would be to learn the general motif changes in key sites that lead to the adoption of multiple states, and exploit that in design.

## Discussion

Over the last 3 years, deep learning has revolutionised protein structure prediction (Baek et al., 2021; Jumper et al., 2021). Given the mantra that protein design is effectively the reverse folding problem, it stands to reason that DL methods should also impact protein design. However, while we have witnessed the rapid growth of DL based methods, much like in protein structure, the question of how to accommodate protein flexibility, particularly their ability to adopt multiple conformational states for function, remains. The explicit inclusion of conformational flexibility and switching properties in protein design has a wide range of biomedical applications. For example, the development of synthetic light-activated ion channels for studying neurological disorders (Beck et al., 2018), the engineering of GPCR biomarkers that trigger on diagnostic ligand association (Adeniran et al., 2018), and the design of highly ligand specific molecular on-switches that mediate CAR T-cell activity (Zajc et al., 2020).

Most existing design methods pivot towards either sequence (Wu et al., 2021) or structure generation (Ovchinnikov and Huang, 2021), with significant strides having been made with both approaches. Some design methods have even pioneered the design of both structure and sequence simultaneously (Tischer et al., 2020; Yang et al., 2020; Anishchenko et al., 2021), which is necessary when designing proteins with multiple fold states. However, it is worth noting that, with few exceptions (Grisoni et al., 2018; Amimeur et al., 2020; Linder et al., 2020; Strokach et al., 2020; Anishchenko et al., 2021; Hawkins-Hooker et al., 2021; Repecka et al., 2021; Anand et al., 2022) most DL design methods lack any experimental validation, relying instead on pure *in silico* examination. Experimental validation is essential to truly validate a network and examine whether they are transferable to other systems.

Coupling the loss of generated structure-sequence topologies to the dynamic fold state of a protein is beyond the capabilities of current generative modelling design algorithms. Nevertheless, we have already observed successful *de novo* design of proteins *via* DL methods and the harnessing of MSA data within the structure prediction field to extract multiple conformations landscapes of a protein from a single sequence given contextual information. Given that conformational landscape optimisation is increasingly employed in design, and the generalisability of MSA-based networks have demonstrated that multiple conformational landscapes can be intimately linked to the same sequence, greater exploitation of MSA in DL-based protein design could yield *de novo* topologies able to adopt multiple conformations based on some stimulus. However, it has been indicated that AlphaFold2 is unable to accurately learn the underlying energy landscape that describes protein folding and function (Saldaño et al., 2021). Thus, future design methods could be assisted by DL work in orthogonal fields, which have shown their ability to predict ensembles of biophysically related states (Jin et al., 2021; Ramaswamy et al., 2021; Tian et al., 2021). Of particular interest are networks where the loss includes explicit physics-based terms (Ramaswamy et al., 2021), which could be seen as an alternative to the MSA bioinformatics approach, offering a more intimate understanding of a protein’s folding landscape during design while ensuring that kinetic pathways are accessible between states.

Over the last few decades, protein design has been based on re-engineering native proteins to alter their functionality. Yet, this restricts our programming of novel function. While there have been pure *de novo* successes, protein design remains a highly complex optimisation problem with the vast space of all possible sequences and structures far outside the known proteome (Huang et al., 2016a) inaccessible to traditional approaches. DL is well suited for these complex tasks, having already revolutionised the structure prediction field. AlphaFold2 and RoseTTAfold present a general solution to the protein folding problem. Protein design, considered the inverse problem, contributes an additional layer of complexity. Rather than just predicting a plausible structure from sequence, comprehensive programmability requires an appreciation of how the sequence, structure and dynamic conformational state of a protein underpin its function. DL is already expanding our design capabilities and knowledge of a dynamic proteome, while the machine learning field itself is undergoing significant and continuous innovation. Leveraging these evolving techniques while improving the exploitation of MSA data or physics-based descriptors could prove key to designing proteins with significant conformational flexibility and thus more advanced functional capacity.
